# Wavefront sensing at X-ray free-electron lasers^1^

**DOI:** 10.1107/S1600577519005721

**Published:** 2019-06-19

**Authors:** Matthew Seaberg, Ruxandra Cojocaru, Sebastien Berujon, Eric Ziegler, Andreas Jaggi, Juraj Krempasky, Frank Seiboth, Andrew Aquila, Yanwei Liu, Anne Sakdinawat, Hae Ja Lee, Uwe Flechsig, Luc Patthey, Frieder Koch, Gediminas Seniutinas, Christian David, Diling Zhu, Ladislav Mikeš, Mikako Makita, Takahisa Koyama, Adrian P. Mancuso, Henry N. Chapman, Patrik Vagovič

**Affiliations:** a Linac Coherent Light Source, SLAC National Accelerator Laboratory, Menlo Park, CA 94025, USA; b European Synchrotron Radiation Facility, CS 40220, F-38043 Grenoble Cedex 9, France; c Paul Scherrer Institut, 5232 Villigen PSI, Switzerland; d Deutsches Elektronen-Synchrotron DESY, Notkestraße 85, D-22607 Hamburg, Germany; e Stanford Synchrotron Radiation Lightsource, SLAC National Accelerator Laboratory, Menlo Park, CA 94025, USA; f European XFEL GmbH, Holzkoppel 4, 22869 Schenefeld, Germany; g RIKEN SPring-8 Center, 1-1-1 Kouto, Sayo-cho, Sayo-gun, Hyogo 679-5148, Japan; h Japan Synchrotron Radiation Research Institute (JASRI), 1-1-1 Kouto, Sayo-cho, Sayo-gun, Hyogo 679-5198, Japan; i Department of Chemistry and Physics, La Trobe Institute for Molecular Science, La Trobe University, Melbourne, Victoria 3086, Australia; j Center for Free-Electron Laser Science, DESY, Notkestraße 85, 22607 Hamburg, Germany; k Centre for Ultrafast Imaging, University of Hamburg, Luruper Chaussee 149, 22607 Hamburg, Germany; l Department of Physics, University of Hamburg, Luruper Chaussee 149, 22607 Hamburg, Germany; m Institute of Physics, Academy of Sciences of the Czech Republic v.v.i., Na Slovance 2, 182 21, Praha 8, Czech Republic

**Keywords:** X-ray free-electron lasers, wavefront sensing, grating interferometry, speckle tracking

## Abstract

Here a direct comparison is made between various X-ray wavefront sensing methods with application to optics alignment and focus characterization at X-ray free-electron lasers (XFELs). Focus optimization at XFEL beamlines presents unique challenges due to high peak powers as well as beam pointing instability, meaning that techniques capable of single-shot measurement and that probe the wavefront at an out-of-focus location are desirable. The techniques chosen for the comparison include single-phase-grating Talbot interferometry (shearing interferometry), dual-grating Talbot interferometry (moiré deflectometry) and speckle tracking. All three methods were implemented during a single beam time at the Linac Coherent Light Source, at the X-ray Pump Probe beamline, in order to make a direct comparison. Each method was used to characterize the wavefront resulting from a stack of beryllium compound refractive lenses followed by a corrective phase plate. In addition, difference wavefront measurements with and without the phase plate agreed with its design to within λ/20, which enabled a direct quantitative comparison between methods. Finally, a path toward automated alignment at XFEL beamlines using a wavefront sensor to close the loop is presented.

## Abstract 2

Here a direct comparison is made between various X-ray wavefront sensing methods with application to optics alignment and focus characterization at X-ray free-electron lasers. Difference wavefront measurements with and without a corrective phase plate agreed with its design to within λ/20, enabling a direct quantitative comparison between methods.

## 1. Introduction   

As more X-ray free-electron lasers (XFELs) come online around the world and the scientific demands of these light sources becomes ever greater, there is an increasing need for the further development of a broad suite of diagnostics (Tschentscher *et al.*, 2006 ▸; Ackermann *et al.*, 2007 ▸; Allaria *et al.*, 2010 ▸; Yabashi *et al.*, 2015 ▸; Bostedt *et al.*, 2016 ▸; Milne *et al.*, 2017 ▸; Ko *et al.*, 2017 ▸). Experiments are typically performed at XFELs for one of two following reasons: to take advantage of the high time resolution they afford in contrast to that available from third-generation synchrotrons and in cases when high X-ray peak intensities are needed (Bostedt *et al.*, 2016 ▸). In the latter case, focus optimization and characterization are especially important. For instance, in order to understand data in nonlinear optics experiments an accurate knowledge of the X-ray focus profile is critical (Fuchs *et al.*, 2015 ▸). In addition, experiments performed with the goal of single-particle imaging benefit from good focusing in order to achieve higher total scattering efficiencies (Aquila *et al.*, 2015 ▸). These experiments drive the need for devices that are sensitive to the X-ray wavefront both for optics alignment and for focus characterization.

Focus characterization is especially challenging at XFELs, for which methods that involve placing a probe close to the focus are either subject to damage (*i.e.* knife-edge, ptychography) or time-consuming (imprints) (Sikorski *et al.*, 2015 ▸). Further, techniques that are capable of measuring in a single shot are critical to account for and characterize the role that spatial jitter can play at these light sources. In this work we describe three related deterministic techniques currently in use at XFELs, all of which make use of information based on near-field diffraction to directly measure the gradient of the X-ray wavefront: single-grating Talbot interferometry, moiré deflectometry and speckle tracking. In Section 3 we describe the requirements and methods behind each technique. In Section 4 we discuss the progress that has been made towards automated alignment of an XFEL beamline and what remains to be done. Finally, in Section 5 we present the results of a direct comparison between the three techniques at the X-ray Pump Probe (XPP) beamline of the Linac Coherent Light Source (LCLS).

## 2. Experimental setup   

We used the monochromatic beam delivered by the dual crystal diamond monochromator at XPP as the platform for our comparison, centered at a photon energy of 9.5 keV (Zhu *et al.*, 2014 ▸). In this comparison we made use of beryllium compound refractive lenses (CRLs) to focus the beam and measured the effects of a phase plate designed to compensate for the lens aberrations using the various techniques (Seiboth *et al.*, 2017 ▸). The CRL stack consists of 20 lenses with 50 µm radius of curvature and 300 µm diameter, resulting in a 340 mm focal length and ∼110 nm full width at half-maximum (FWHM) at focus in the absence of aberrations. The corrective phase plate was fabricated using 3D microprinting in nanoscribe IP-S photoresist and its design is based on a model of the characteristic shape error of the lenses described above, without accounting for any imperfections of this particular lens stack.

**Figure 1:**
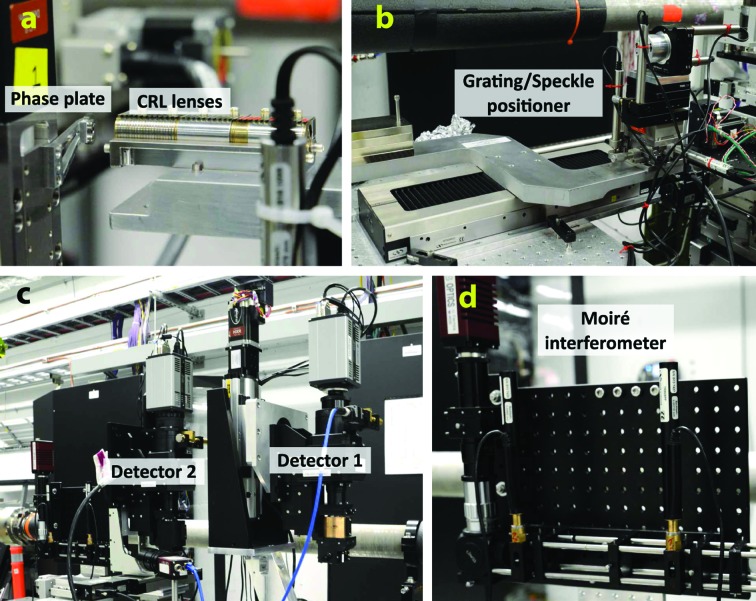
Photographs of the experimental setup. The X-ray beam was focused by a set of beryllium CRL lenses (*a*) and a phase plate with horizontal and vertical degrees of freedom was placed downstream of the CRL optics. The grating/speckle phase modulators were mounted on a motorized positioner with long travel range along the optical axis (*b*). Two indirect X-ray detectors (*c*) with YAG scintillating screens were placed further downstream at approximately 2 m distance from the focus, and the compact moiré interferometer was mounted as the last part of the setup (*d*).

A custom setup designed to accommodate the three available techniques was designed and implemented as shown in Fig. 1 ▸. The CRL stack is motorized with five degrees of freedom allowing it to be aligned onto the X-ray beam’s optical axis and the phase plate is motorized with horizontal and vertical degrees of freedom [Fig. 1(*a*) ▸]. The single-grating interferometry and speckle tracking techniques share a mounting platform for the gratings and speckle membranes, with a kinematic mount providing the ability to swap between gratings and membranes in a reproducible manner [Fig. 1(*b*) ▸]. Two scintillator-based indirect X-ray detectors (Optique Peter microscopes) are placed further downstream at a fixed distance from the focus, but with motorized travel in the horizontal and vertical directions [Fig. 1(*c*) ▸]. The detector labeled as Detector 1 is partially transparent and was only used for the speckle tracking measurements. Detector 2 was used for both single-grating interferometry and speckle tracking measurements. Finally, the moiré interferometer, which is self-contained, is located just downstream of Detector 2 [Fig. 1(*d*) ▸].

## 3. Wavefront sensing techniques   

The past couple of decades have seen major advances in X-ray wavefront sensing (Naulleau *et al.*, 2000 ▸; David *et al.*, 2002 ▸; Rutishauser *et al.*, 2011 ▸, 2012 ▸; Matsuyama *et al.*, 2012 ▸; Kayser *et al.*, 2014 ▸, 2016 ▸; Berujon *et al.*, 2015 ▸; Assoufid *et al.*, 2016 ▸; Liu *et al.*, 2018 ▸), that have also benefited from the long history of wavefront sensing methods in the visible region of the spectrum. All three techniques discussed here make use of near-field diffraction for direct measurement of the wavefront gradient. As will be seen in the following, each technique has various advantages and disadvantages, but in the end all give similar, accurate results.

### 3.1. Single-phase-grating Talbot interferometry   

**Figure 2:**
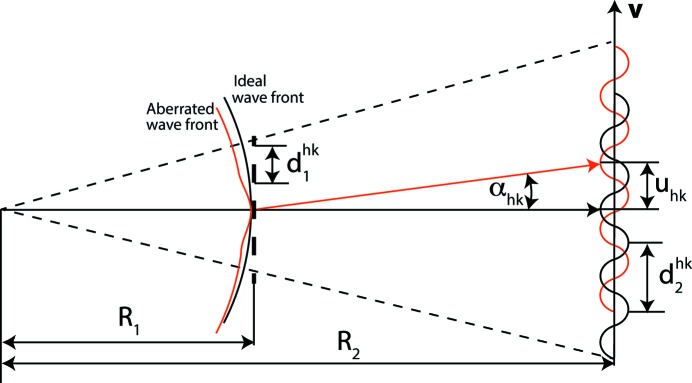
Operation principle of the single-phase-grating method for wavefront shape measurement; **v** is the unit vector in the direction of **q**_*hk*_.

When the periodic modulator, amplitude or phase grating, is illuminated with a partially or fully coherent X-ray wavefield, the so-called self-images can be measured downstream of the modulator. We refer to these measurements as interferograms. The interferograms are measured at discrete distances. In the case of an absorption grating, strong modulations occur at Talbot distances, but in the case of a phase grating strong modulations occur at intermediate distances called fractional distances. The contrast of the interferograms is maximized at the planes perpendicular to the optical axis (Suleski, 1997 ▸) given by distances
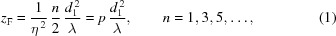
where η = 1 in the case of a π/2 phase grating and η = 2 in the case of a π phase shifting grating; *p* is a generalized parameter that we refer to as the Talbot order, *d*_1_ is the period of the grating, and λ is the X-ray wavelength. If the conical illumination (divergent beam generated for example by focusing optics) is applied, the fractional distance *z*_F_ will be scaled by the geometrical magnification *M* (Fresnel scaling theorem; Paganin & Press, 2006 ▸),
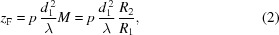
where *R*_2_ is the distance of the image plane to the focal plane and *R*_1_ is the distance of the grating to the focal plane (see Fig. 2 ▸).

The pitch of the detected interferogram *d*_2_ will be equally scaled by the magnification, 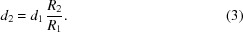
Combining scaled equation for the fractional Talbot distance (2) and with geometric relation for the distances, 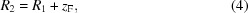
we obtain the convenient equation reported by Yashiro *et al.* (2009 ▸),
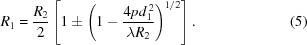
Equation (5) has two solutions for a given Talbot order. The solution with the plus sign means that the grating position is closer to the imaging plane, and that with the minus sign refers to the grating placed closer to the focal plane. If the latter option is used, high magnification and angular sensitivity can be achieved.

The interferogram measured downstream of the phase grating has the same spatial structure as the original phase grating with the best resemblance at the fractional Talbot distances. Any 2D periodic structure can be expressed using notation known from crystallography as
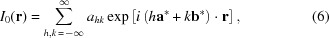
where 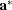 and 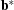 are the reciprocal lattice vectors and *h*, *k* are integers (Miller indices). If we set real space vectors of the periodic pattern |**a**| = |**b**| and the angle between them equal to π/2 we obtain a checkerboard lattice.

The wavefront shape with residual distortions (aberrations), that we want to recover, is modulated on this periodic pattern at the detector plane as a vectorial displacement field **u**(**r**) of the ideal pattern (Fig. 2 ▸). The displacement field is given approximately by
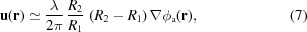
where ϕ_a_(**r**) refers to the aspheric component of the wavefront phase. This is an approximation in the case of large grating shear, which is discussed further below. The intensity distribution at the detector plane in the presence of a distortion field is given as
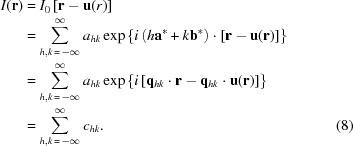
We call the coefficients *c*_*hk*_ complex visibility containing amplitude *a*_*hk*_ and the displacement field is located around the corresponding carrier fringe with frequency **q**_*hk*_. The amplitude coefficients *a*_*hk*_ vary along the optical axis and the local maxima are located at the corresponding fractional Talbot distances. It is also clear that around each carrier peak the measured displacement only has a component in the direction of the vector **q**_*hk*_. Therefore, in order to extract the full displacement field, at least two carrier peaks with reciprocal vectors not parallel to each other need to be analyzed. The zero-order term *c*_00_ = *a*_00_ represents the unperturbed intensity of the background. This term is used to obtain the amplitude of the wavefront. However, due to the fact that this method is in its principle shearing interferometry, the zero-order term contains shifted replicas resulting in blur of the demodulated amplitude (Servin *et al.*, 2007 ▸). This can be neglected if the shear is smaller than the resolution element (period of the fringe). In the case of larger shear, the amplitude cannot be reconstructed uniquely and the extracted phase during the demodulation is the phase difference between two shifted beams at the detector plane, which is recovered using the Fourier method (Takeda *et al.*, 1982 ▸; Servin *et al.*, 2007 ▸). The differential phase can be obtained uniquely by applying the shear transfer function on the phase difference data (Elster & Weingärtner, 1999 ▸), but due to poles of the shear transfer function regularization has to be applied. An additional consideration in the choice of grating shear is the trade-off between sensitivity and dynamic range. As shear (and thus sensitivity) is increased, the fringes may begin to blur together in the case of large aspheric wavefront slopes. Further analysis of the influence of the shearing ratio on the X-ray wavefront data will be the scope of our future work.

**Figure 3:**
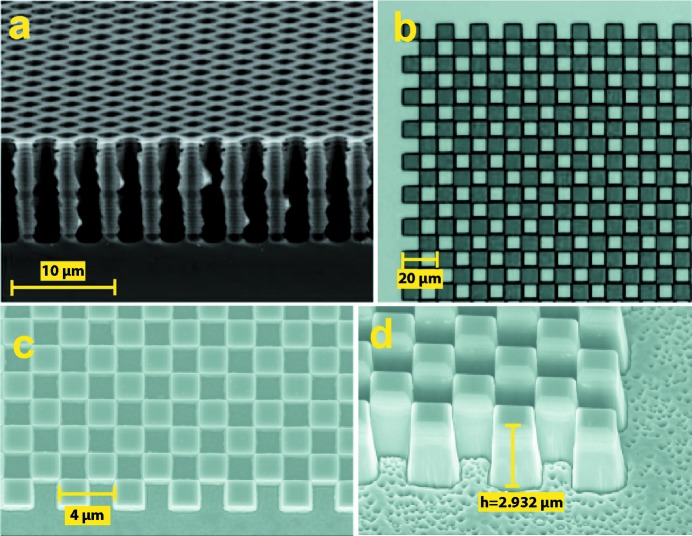
The π and π/2 phase gratings used in the single-phase-grating setup. (*a*) Si π grating, *d*_1_ = 2.725 µm, height h = 11 µm; (*b*) Si π grating, *d*_1_ = 14.142 µm, *h* = 12 µm; (*c*,*d*) CVD diamond π/2 phase grating, *d*_1_ = 2.282 µm, *h* = 2.932 µm.

The checkerboard π and π/2 phase gratings used for this study are shown in Fig. 3 ▸. The Si grating [Fig. 3(*a*) ▸] has a pitch along the diagonal 2.725 µm, the height of the structure is 11 µm giving π phase shift at 9 keV and was patterned by reactive ion etching into Si substrate (Rutishauser *et al.*, 2011 ▸). A second Si grating [Fig. 3(*b*) ▸] has a pitch along the diagonal 14.142 µm, the height of the structure is 12 µm giving π phase shift at 9.5 keV. The patterning was performed with electron beam lithography into a hydrogen silsesquioxane resist, which was used as a mask to etch into the silicon (Liu *et al.*, 2018 ▸). The last grating [Figs. 3(*c*) and 3(*d*) ▸] is a diamond grating with a pitch along the diagonal of 2.828 µm and structure height of 2.932 µm, giving π/2 phase shift at 8 keV. The grating has been patterned by electron beam lithography of chemical vapor deposition (CVD) diamond membrane (Makita *et al.*, 2017 ▸).

**Figure 4:**
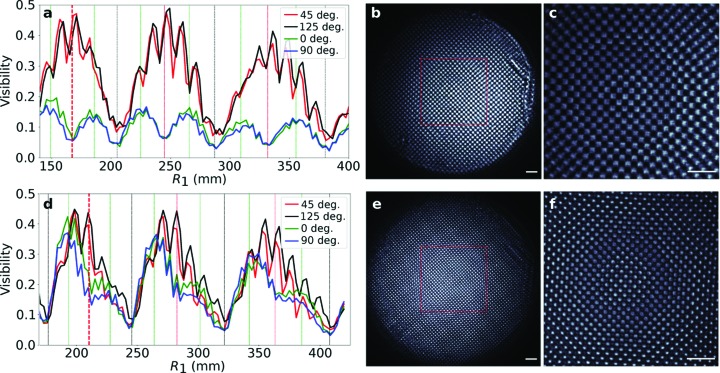
Comparison of visibility variation along the optical axis for basic (45° and 125° direction) and mixed harmonics (0° and 90° direction) for π/2 diamond (*a*) and π Si (*d*) phase checkerboard gratings. The pitch in the diagonal direction was 2.8 µm for the diamond grating and 2.725 µm for the Si grating. Vertical lines in the graphs represent the theoretical positions for the Talbot distances (black) and fractional Talbot distances for basic (red) and mixed (green) harmonics. The interferograms (*b*,*c*,*e*,*f*) shown are taken from the first maxima for the basic harmonics. The scale bar represents 100 µm. For the analysis of the visibility the area marked with red squares in (*b*) and (*e*) was used. The locations and sizes of these regions of interest were chosen for visibility analysis based on the nearly uniform illumination within these areas.

To understand the performance of the single-grating interferometer we performed scans of the phase gratings along the optical axis in the ‘minus geometry’ in equation (5) and recorded a series of images. For fair comparison we used two types, π silicon and π/2 diamond phase shifting gratings, shown in Figs. 3(*a*) and 3(*d*) ▸, and analyzed the visibility of the basic and mixed harmonics (Fig. 4 ▸). The detection was made with a scintillator-based, 4.5× microscope (Optique Peter white beam microscope with Andor Zyla 5.5 camera). The distance from focus to grating is displayed on the *x*-axis of the plots in Figs. 4(*a*) and 4(*e*) ▸ for the two gratings, and the distance from focus to detector was fixed at 1.925 m. Figs. 4(*a*) and 4(*e*) ▸ show the visibility of the basic harmonics (diagonal direction) and mixed harmonics (0° and 90°) direction of the two gratings. Interestingly the mixed harmonics for the π grating are reaching nearly the same visibility as the basic harmonics while in the case of the π/2 grating the mixed harmonics are suppressed. Therefore if one wants to have sensitivity in more than two directions using checkerboard gratings the π grating is a more suitable choice. The short-period oscillations on the visibility curves especially for basic harmonics are caused by optical power flowing to higher orders.

Further measurements were made using the 14.1 µm π phase silicon grating [Fig. 3(*b*) ▸]. This grating was placed at 478 mm from the focus, with the detector again located at 1.925 m from the focus. These measurements were used for the phase plate alignment, discussed in Section 4, and for comparison with the speckle tracking method, discussed in Section 5.

**Figure 5:**
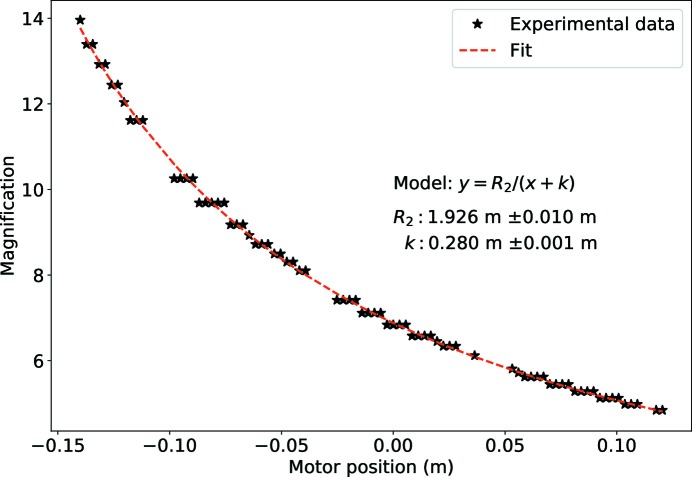
Distances calibration of Si π-phase shifting grating with diagonal pitch size 2.725 µm.

The precise grating-to-focus and focus-to-detector distances have been calibrated using the longitudinal scan of the grating along the optical axis. For each position the fringe size in the given direction has been measured and the magnification was determined from the known grating geometry. Data where visibility for the given harmonic was below 15% have not been considered as the error in determination of the fringe size was high. The magnification curve has then been fitted by least-squares method. An example for the distances calibration of the Si π phase shifting grating, with diagonal pitch size 2.725 µm, using the 45° oriented diagonal fringe is shown in Fig. 5 ▸.

This is the most important calibration step; however, to obtain more accurate results any detector aberrations can be mapped as well. This can be done by, for example, placing an absorbing grid directly in front of the scintillator or by scanning the detector in directions perpendicular to the beam. The tilt of the grating with respect to the detector (and beam) can be recovered via a rotational scan of the grating around the optical axis.

### 3.2. Dual grating Talbot interferomety (moiré deflectometry)   

**Figure 6:**
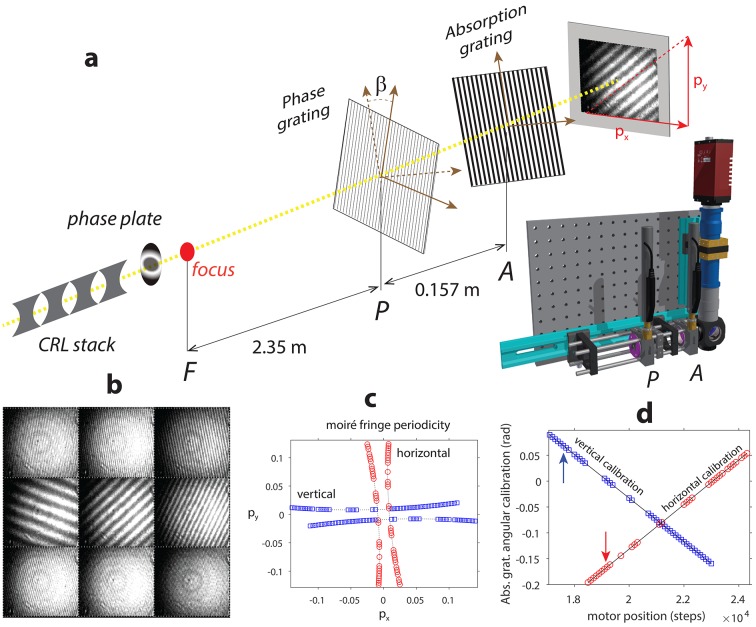
(*a*) Schematic layout of the moiré XGI experimental setup at the SLAC XPP beamline. The XFEL pulses with 9.5 keV photon energy are focused with a stack of 20 compound refractive lenses followed by a corrective phase plate for diffraction-limited focusing. The grating interferometer was installed further downstream, consisting of phase (*P*) and absorption (*A*) gratings. Red arrows indicate moiré fringe periods *p*_*x*_ and *p*_*y*_ extracted via two-dimensional Fourier analysis (see text). (*b*) Selected moiré interferograms measured by rotating the absorption grating; (*c*) with extracted moiré fringe frequencies *p*_*x*_ and *p*_*y*_ in the horizontal and vertical directions for calibration. (*d*) Angular calibration of the moiré XGI, vertical arrows indicate the relative β offset between the two gratings indicated in panel (*a*).

The next at-wavelength wavefront diagnostic method discussed in this report is the X-ray grating interferometry (XGI) method, realized for measurements in the moiré mode. The wavefront properties in shot-to-shot LCLS SASE operation, propagated to a pair of hard X-ray offset mirrors at the XPP beamline, have already been investigated in moiré mode (Rutishauser *et al.*, 2012 ▸). Here we describe a similar setup in which the spatially coherent radiation was further monochromated with a double-crystal monochromator to 9.5 keV and focused by means of a set of compound refractive lenses as schematically depicted in Fig. 6(*a*) ▸. The instrument, seen in Fig. 1(*d*) ▸ ▸, developed at the Swiss Light Source X05DA Optics beamline (Flechsig *et al.*, 2009 ▸), is a modular, compact, transportable and self-contained moiré XGI setup. In the moiré mode the incident X-ray beam is diffracted by periodic, micrometre-sized binary structures just like in single-phase-grating interferometry discussed in Section 3.1. The X-ray wavefronts propagating through the grating have small shear angles and create an interference pattern. Due to the Talbot effect, the interference has maximum contrast at certain, discrete distances downstream of the first grating. These are the locations where the diffraction orders overlap and interferences occur creating self-images of the grating at specific distances called fractional Talbot distances. The best diffraction efficiency of the diffracting grating into the ±1st diffraction order is obtained with binary π-phase shifting gratings and a duty cycle of 0.5. Maximum contrast for the constructive interference pattern at fractional Talbot orders depends on the wavelength and periodicity of the phase grating. Contrary to single-phase-grating Talbot interferometry, a second grating is inserted in front of the detector and used as a transmission mask (which has a period matching the one of the interference pattern). The interference pattern produced by the first grating at the distance *PA*, where the second absorption grating is located, is magnified from the expected self-referenced image by the design magnification of the interferometer [*M* = (*FP* + *PA*)/*FP*]. We note that the period of the second grating was designed to match the self-image of the phase grating in the diverging beam (Rutishauser, 2013 ▸). The moiré XGI experiment was optimized for 9.5 keV photon energy by considering the available space downstream of the CRL stack. For divergence matching a periodicity of 3.75 µm was used for the first grating *P* and the absorption grating had the same duty cycle with periodicity of 2 µm. By optimizing the spatial illumination and visibility of moiré interferograms at the detector plane we found that the 11th fractional Talbot order gave best results, while maintaining a compact 15.7 cm distance between the two gratings. more detailed description of the moiré XGI setup optimized for various setups has been given by Krempaský *et al.* (2018 ▸).

**Figure 7:**
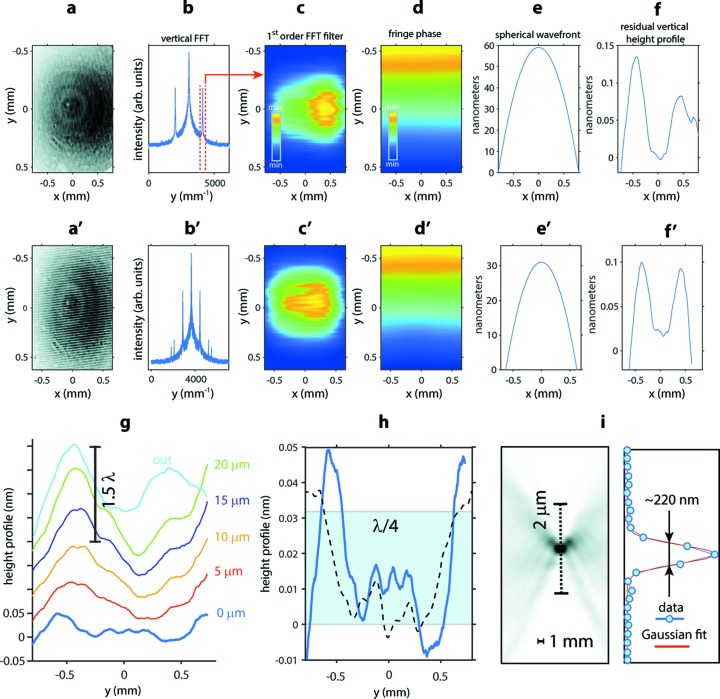
Flowchart of the moiré XGI algorithm with horizontally (*a*–*f*) and vertically (*a*′–*f*′) aligned line gratings (see text). (*g*) Lineout stack of wavefront errors in the vertical direction showing gradual improvement as the phase plate is moving toward the central position (0 µm). (*h*) Comparison of wavefront errors between moiré XGI [bottom line in (*g*)] and single-phase-grating Talbot interferometry (dashed line) at the central phase plate position (0 µm). The blue rectangle is marking the expected λ/4 wavefront error improvement compared with ∼1.5λ denoted by vertical segment in panel (*g*). (*i*) Vertical through-focus and focal plane intensity profile retrieved using amplitude and phase back-propagation. The amplitude profile extends over the first-order amplitude peak center in vertical direction, the related height profile is seen in panel (*h*). The vertical beam waist profile with ∼220 nm FWHM was extracted along the dashed segment.

Figs. 6(*b*)–6(*d*) ▸ summarize the calibration of the moiré XGI which in our case is based on rotating the phase and absorbing gratings with respect to each other around the optical axis. The moiré patterns seen in Fig. 6(*b*) ▸ are integrated over 100 shots to achieve sufficient moiré fringe visibility. For a quantitative analysis Fig. 6(*c*) ▸ shows a calibration scan by recording the moiré patterns at varying angular orientations of the absorption grating. The fringe periods *p*_*x*_ and *p*_*y*_ in the horizontal and vertical directions are extracted via two-dimensional Fourier analysis. For π-phase shifting gratings the interference fringe period and the absorption grating period are connected through orientation angles β denoted in Fig. 6(*a*) ▸, that can be obtained from a minimization procedure of data in Fig. 6(*c*) ▸ (Rutishauser, 2013 ▸; Kayser *et al.*, 2017 ▸). More specifically, the phase and absorption grating absolute angles in our case were determined by keeping the phase grating fixed; vertical arrows in Fig. 6(*d*) ▸ refer to absolute absorption grating angles in measurements summarized in Fig. 7 ▸. We note that the shape of the curves in Fig. 6(*c*) ▸ confirm that all the wavefronts discussed below relate to focusing beams (Rutishauser, 2013 ▸). A more detailed description of the XGI-based moiré interferometry has been given by Pfeiffer *et al.* (2005 ▸), Weitkamp *et al.* (2005 ▸), Wang *et al.* (2011 ▸), Rutishauser (2013 ▸) and Kayser *et al.* (2017 ▸).

Once the phase grating absolute angle and absorption grating offset β is derived from the calibration scan, a full wavefront characterization can be determined from single images which we further inspect in the vertical [Figs. 7(*f*)–7(*h*) ▸] and horizontal directions [Fig. 7(*a*′)–7(*f*′) ▸], respectively. The panels summarize the flowcharts of the computational algorithm. First a moiré interferogram in panel (*a*,*a*′) is Fourier filtered in panel (*c*,*c*′) (Takeda *et al.*, 1982 ▸), vertical dashed lines in panel (*b*) indicate the filter width used to retrieve the Fourier transform first-order component. Panels (*d*,*d*′) extract the moiré fringe phase images from which the wavefront propagation (deflection) angle in the direction perpendicular to the grating lines is retrieved. Next the wavefront phase is obtained by integration in the corresponding directions in the detector plane. Finally, panels (*e*,*e*′) show the spherical fit of the wavefront phase and (*f*,*f*′) show the wavefront aspherical shapes retrieved by subtracting a second-order polynomial. For a quantitative description of the wavefront distortions data are converted to height profiles by multiplying the phase distortions by λ/2π. The height profiles indicate that the distortions are comparable with the X-ray wavelength (∼0.13 nm) in both directions. Both lineouts have a pronounced dip in the middle typical of wavefront distortions around the center of the CRL exit pupil (Seiboth *et al.*, 2017 ▸), consistent with the wavefront profile measured by means of speckle tracking (Fig. 9) discussed below.

Next we discuss the reduction of wavefront distortions by correcting the residual spherical aberration of the whole CRL stack by a corrective phase plate. Stacked lineouts in Fig. 7(*g*) ▸ show gradual alignment of the CRL exit pupil with a diameter of 300 µm. The top lineout labeled ‘out’, also seen in panel (*f*), corresponds to a completely removed phase plate. The remaining lineouts represent gradual improvements of the wavefront distortions as the phase plate is shifting toward the central position (0 µm). For qualitative and quantitative comparison with single-grating Talbot interferometry summarized in Fig. 10(*b*), a comparison of the wavefront distortions between the two methods at the central position is shown in Fig. 7(*h*) ▸. It turns out that the small differences between the lineouts are mainly due to backlash limitations of the motorized stage (∼8 µm). Indeed, as seen in Fig. 10(*b*), the optimal phase plate position was found at 2 µm. These experimental observations confirm that mechanical alignment of the CRL stack is critical for achieving diffraction-limited focusing.

A full wavefront characterization from a single moiré interferogram implies that both the amplitude and phase information are determined: the first is proportional to the intensity profile of the beam and the second is related to the wavefront’s gradient. Both quantities define the complex electric field of the X-ray beam, which we next back-propagate to the focus. Fig. 7(*i*) ▸ shows the retrieved through-focus and focal plane intensity profiles in the vertical projection. With the phase plate inserted, the average Gaussian fit of the beam caustic indicates a vertical focus FWHM around 220 nm. Compared with single-shot 2D focus retrieval based on single-grating Talbot interferometry seen in Fig. 11(*f*), the beam waist from 100 FEL pulses is ∼50 nm larger. Such a discrepancy can be attributed to subtle mechanical misalignment of the CRL stack mentioned above, as well as data averaging from 100 FEL pulses.

To conclude, the wavefront analysis between moiré and single-grating Talbot grating interferometry shows consistent improvement of the wavefront height profile from ∼1.5λ down to ∼λ/4. Given the fact that the phase plate conceptual design for a stack of 20 CRLs was based on an average lens model (Seiboth *et al.*, 2018 ▸) and the refractive index of the cured IP-S photoresist is only estimated, not measured, the performance of the phase plate correction is consistent with expected results. The presented moiré XGI setup thus provides a valuable at wavelength diagnostics tool for qualitative and quantitative, spatially resolved X-ray wavefront metrology at synchrotron radiation beamlines or XFEL endstations.

### 3.3. Speckle tracking   

X-ray speckle tracking is a wavefront sensing technique that can accurately retrieve the wavefront gradient of the X-ray beam by making use of random intensity modulations (Bérujon *et al.*, 2012 ▸). A phase object with small features is used to generate two near-field speckle images, which are then analyzed using numerical cross-correlations and template matching algorithms to identify the angular displacement of small subsets from the first image into the second. Given that in the optical near-field region the distortion of the speckle pattern depends only on the wavefront propagation (Cerbino *et al.*, 2008 ▸), the modulation displacements are proportional to the wavefront *W* (and phase ϕ) gradients through the equations
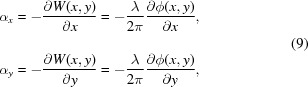
where α_*x*, *y*_ are measured deflection angles. Finally, the wavefront is recovered by numerically integrating these two orthogonal gradients using either a least-square minimization algorithm (O’Leary & Harker, 2008 ▸, 2012 ▸) or a fast Fourier transform based algorithm like the one of Frankot & Chellappa (1988 ▸).

Note that although a random pattern used to modulate the wavefront contains many frequencies in its Fourier spectrum, not all of them are recorded onto the detector. This is again due to the Talbot effect which, for a fixed distance, acts as a spectral filter with a **q**-frequency modulation 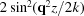.

**Figure 8:**
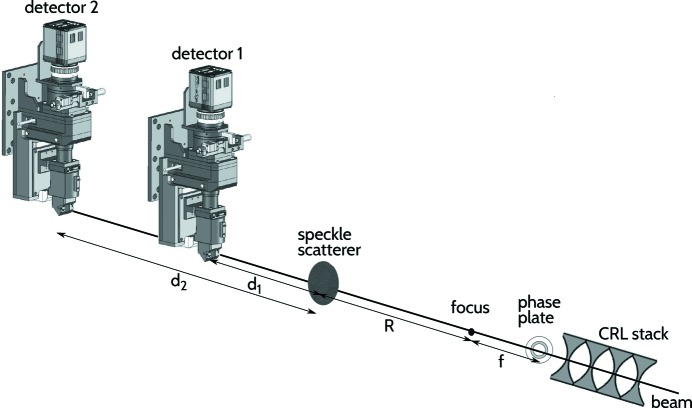
Sketch of the experimental setup used to perform X-ray speckle tracking in absolute and differential mode. A speckle scatterer and two synchronized indirect X-ray detectors, the first one being semi-transparent, are aligned along the beam. For our experiment, the distances were fixed to: *d*_1_ = 905 mm, *d*_2_ = 1325 mm, *R* = 590 mm and *f* = 308 mm (see text for further details).

A speckle-based wavefront sensor such as the one used here is adequate for measuring hard X-rays with photon energies between 8 keV and 40 keV and can be used in two different regimes: the differential and the absolute modes. The setup for differential mode measurements only requires a speckle scatterer and an X-ray detector to record consecutive speckle images. This can be used to track wavefront changes over time or to isolate the contribution to the wavefront distortion generated by an individual optical element, *e.g.* a lens or a phase plate. The absolute mode in contrast can be used to measure the true effective wavefront of the beam by using two speckle images acquired simultaneously at two different distances from the speckle scatterer (Berujon *et al.*, 2015 ▸). The corresponding setup is illustrated in Fig. 8 ▸, where distances were chosen taking into account the magnification of the system while also allowing sufficient propagation so as to obtain the desired sensitivity. The method itself does not place stringent restrictions on the distances between the speckle generator and the detector(s). One effect of changing these distances in the setup will be to vary the resolvable pattern frequencies. In any case, within the near field the speckle pattern will always contain useful frequencies that can be used for modulation. Typically, we set the distance *d*_2_ at a value roughly equal to (*sN*)^2^/λ, where *s* represents the pixel size, *N* the average speckle size in pixels, and λ the photon wavelength, which corresponds to a Fresnel number *F* ≃ 1, at the limit of the near-field regime. The absolute mode is experimentally more demanding for single pulse characterization since it requires two separate synchronized and well aligned indirect X-ray detectors. The first of these detectors must be semi-transparent to X-rays in order to allow sufficient flux to fall onto the second detector. Our technical solutions consisted of a first detector based on a thin 25 µm YAG:Ce scintillator and a pierced silica substrate mirror with a 1.5 mm hole, ensuring a transparency of over 25% at 9.5 keV. As we shall demonstrate, this speckle-based wavefront sensing approach can be performed with sub-wavelength accuracy for single pulse measurements.

**Figure 9:**
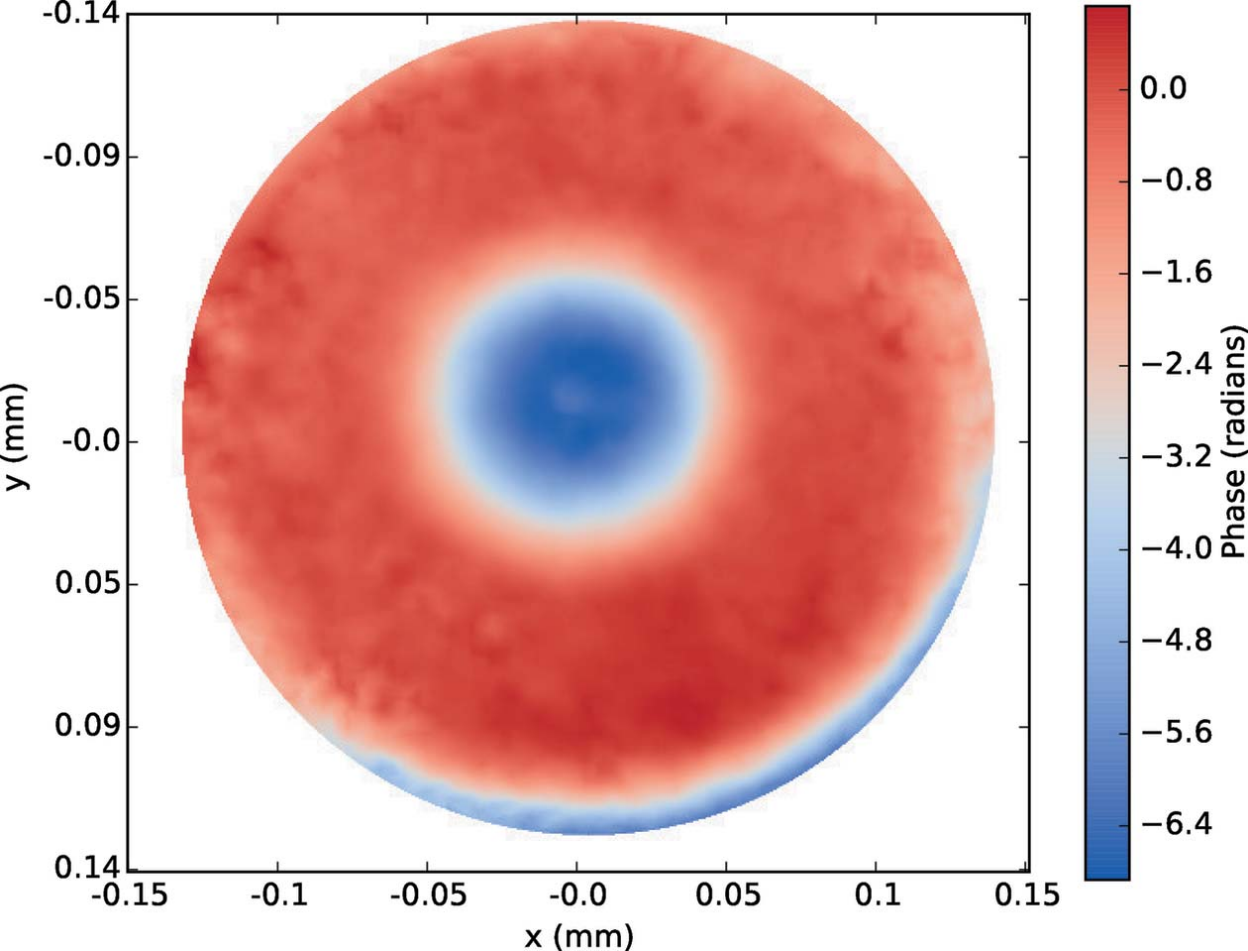
Recovered profile of the full phase plate, based on X-ray speckle tracking in differential mode. This shows the full contribution of the phase plate to the wavefront, spherical term included. Note that the plate was not well centered at this time.

Measurements were performed in the differential mode, collecting speckle images with and without the phase plate present in the beam. By numerically comparing these images, the wavefront contribution of the phase plate is recovered, as shown in Fig. 9 ▸. Note that in this example the phase plate was not yet perfectly centered. The phase plate wavefront profile recovered here is compared with the phase plate design curve and with the profile obtained using single-grating interferometry in Fig. 12. Given that the phase plate was not yet perfectly centered at the time when the speckle measurements were performed, a circular mask with a 100 µm radius was applied to the data in order to ensure that a full 360° azimuthal average could be computed for every point of the resulting profile.

## 4. Toward automated beamline alignment   

Maintenance of beamline alignment in cases where tight focusing is required poses a significant challenge at XFEL beamlines. In these cases, alignment typically needs to be checked at the beginning of every shift. Wavefront sensors that are integrated into the beamline have the potential to make a major impact on both focusing quality as well as operational efficiency. Automatic processing of the wavefront measurement can be applied to close the loop on mirror alignment. Here we show a first step toward this goal, with fast wavefront monitoring enabling real-time interactive alignment.

The first step toward automation is real-time data processing. In some cases, each optical degree of freedom can be associated directly with a single aspect of the wavefront. In general the effects of each degree of freedom on the wavefront can be calibrated with the wavefront sensor in order to automatically optimize the wavefront; this general approach has been used for decades to optimize telescope and visible laser wavefronts (Wallner, 1983 ▸).

**Figure 10:**
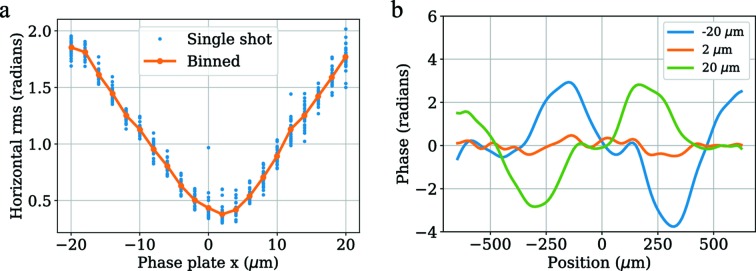
Illustration of real-time processing for phase plate alignment. (*a*) An example of what can be seen live during the measurement. The root mean square of 1D horizontal lineouts of the aspheric phase are used to obtain a measure of the aberrations in the horizontal direction, which in this case are strongly affected by the relative alignment between the phase plate and CRLs. (*b*) Single-shot horizontal aspheric phase lineouts are shown for the first, optimal and last positions in the scan, which were −20 µm, 2 µm and 20 µm, respectively.

Here, we use the wavefront sensor to align a phase plate interactively with visual feedback. In this case the wavefront error relative to phase plate position is mostly decoupled between the horizontal and vertical degrees of freedom. The horizontal position can be optimized by minimizing the r.m.s. aspheric wavefront error along a 1D horizontal lineout of the beam, as shown in Fig. 10(*a*) ▸; this is a reproduction of a plot that can be viewed in real time at LCLS. The wavefront lineouts (with spherical term removed) at three different positions of the scan (endpoints and optimized position) are shown in Fig. 10(*b*) ▸. 2D reconstructions of the wavefront phase and resulting focal spot during this scan are shown in Video S1 of the supporting information.

## 5. Phase plate alignment and comparison   

**Figure 11:**
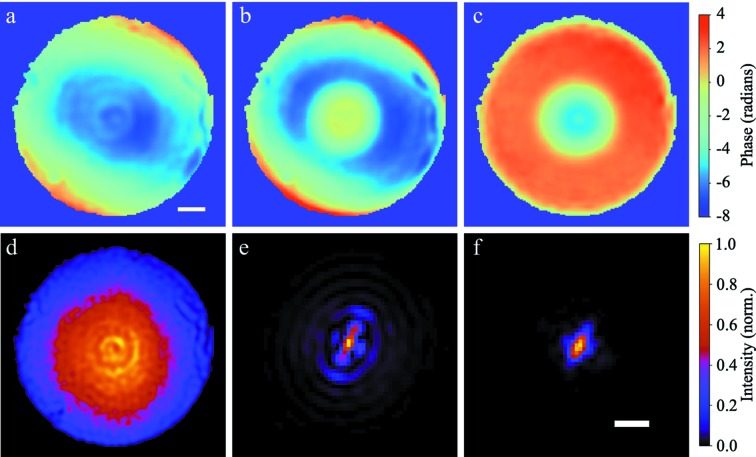
2D wavefront retrieval with and without the corrective phase plate, based on single FEL shots and using a π-phase checkerboard grating with 14.1 µm diagonal pitch. (*a*) Wavefront with phase plate aligned, with scale bar corresponding to 200 µm at the detection plane. (*b*) Wavefront with phase plate removed. (*c*) Recovered profile of phase plate, based on subtraction of (*b*) from (*a*). The colorbar at the right of (*c*) is shared among all three phase profiles. The intensity of the beam can also be extracted from the Talbot image (*d*), which in combination with the retrieved wavefront can be used to obtain profiles of the focus, without (*e*) and with (*f*) phase plate correction. From a visual inspection of (*e*) and (*f*) it can be seen that the phase plate removes most of the fourth-order spherical aberration, but does not compensate for the astigmatism that likely comes from the monochromator crystals. The scale bar in (*f*) corresponds to 500 nm and is shared with (*e*), and the colorbar at right of (*f*) is shared between (*d*)–(*f*).

**Figure 12:**
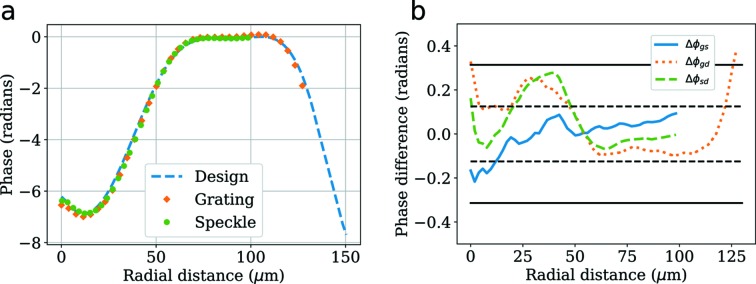
(*a*) The radial profile of the phase plate was measured using both single-grating interferometry and X-ray speckle tracking in differential mode by making measurements of the wavefront, with and without the phase plate inserted, and performing an azimuthal average of the resulting 2D profile [see Fig. 11(*c*) ▸]. Since care was taken to process data with and without the phase plate in exactly the same way, the only fit parameter is a scaling of the radial coordinates. (*b*) A quantitative comparison can be made by examining the phase difference between the curves from (*a*). The curves labeled Δϕ_gs_, Δϕ_gd_ and Δϕ_sd_ refer to differences between grating and speckle, grating and design, and speckle and design, respectively. The region between the solid horizontal lines corresponds to agreement within ±λ/20 and the region between the dashed horizontal lines corresponds to within ±λ/50.

During a single experiment at the LCLS, direct comparisons were made between the three techniques discussed in Section 3. A convenient accuracy comparison can be made via the phase profile of the phase plate used to improve the focus, since the phase plate was manufactured with a known profile. This measurement can be made simply by subtracting the wavefront with the phase plate inserted from the one without it [see Figs. 11(*a*)–11(*c*) ▸ for the corresponding measurements with a π-phase checkerboard grating]. A quantitative comparison between wavefront measurement techniques can be made by comparing radial lineouts of the phase profiles, which is shown in Fig. 12 ▸. The lineouts as measured with the single-grating technique and the speckle tracking method are overlaid with the design in Fig. 12(*a*), with their differences shown in Fig. 12(*b*) ▸. As can be seen, the two measurements agree within ∼λ/50 and they both agree with the design to within λ/20, with the main difference near a radial distance of zero. This disagreement may be due to the slightly lower resolution of the grating technique. The feature at the center of the phase plate design has FWHM ∼10 µm and the sampling period of this particular grating interferometer is ∼7 µm at the plane of the phase plate.

The clear improvement in focus quality can be seen when comparing the retrieved focus for both cases in Figs. 11(*e*) and 11(*f*) ▸. While residual astigmatism, likely due to the diamond crystal monochromator, is still apparent, the phase plate removes the majority of the higher-order aberrations.

## 6. Conclusions   

Here a direct comparison between various wavefront sensing techniques available to hard XFELs is made, with evidence that they all provide accurate and detailed information. This is demonstrated based on comparison of the wavefront itself as well as difference data obtained with and without a corrective phase plate. There are various trade-offs between all three techniques discussed here. The clearest difference lies in the scattering media. Both grating techniques rely on a phase grating (*e.g.* checkerboard or hexagonal structure) and the speckle tracking method relies on a more general phase object such as sandpaper or other fine-grained objects. All three methods require a relatively high resolution image of the beam downstream of the scatterer, which typically means using a scintillator-based indirect X-ray imaging system as was used in this work. However, in the dual grating technique the downstream analyzer grating is used to generate a low-frequency beat pattern (moiré) prior to detection such that the technique does not rely as heavily on detector resolution as do the single-grating and speckle tracking techniques. In addition, the grating geometry and design can be optimized in such a way to result in a very compact device, with small distances between gratings and detector. All three techniques can be used to obtain the wavefront on a single-shot basis, but as discussed in Section 3.3 a second downstream detector is required in the speckle tracking case. In addition, whereas grating interferometry relies on the quality of the grating fabrication for its accuracy, the speckle tracking technique makes use of simple scattering materials and a more complex algorithm to achieve the same accuracy, typically with slightly higher resolution. If an appropriate phase modulator is used, such as a diamond phase grating, the photon energy range in the case of the single-grating setup can be pushed down to ∼4 keV. For the other two techniques the lower photon energy boundary is limited by the pure transparency of the first detector in speckle tracking technique and absorption grating damage threshold in moiré deflectometry resulting in a ∼8 keV lower boundary. Finally, when one wants to retrieve the absolute profile of the focus a measurement of the beam’s amplitude in addition to wavefront must also be made. The amplitude information is only readily available in the case of either single or dual grating interferometry when the grating shear is small (*e.g.* of the order of the fringe spacing) and comes at the expense of reduced sensitivity to wavefront aberration. These trade-offs can be considered when making a decision on which measurement scheme should be used at a given beamline.

Grating interferometry and speckle tracking are excellent candidates for *in situ* XFEL diagnostics due to their operation out of focus, thus avoiding damage, and their ability to provide single-shot information. We have shown here that it is possible to use these techniques for real-time optics alignment, which will enable automated beamline alignment and more accurate focus characterization in the future. As the analysis for the grating data relies on several single-step numerical operations such as Fourier filtering and line or surface fitting, the high-speed implementation on, for example, graphical computing units (GPUs) or field-programmable gate arrays (FPGAs) will be possible. This will provide a further step towards implementation of automatic feedback systems and real-time data display. More frequent use of this capability at XFELs will lead to more efficient use of beam time as well as providing additional information for experiments requiring challenging data analysis.

## Supplementary Material

2D reconstructions of the wavefront phase and resulting focal spot. DOI: 10.1107/S1600577519005721/xl5031sup1.mp4
